# Optimizing Cerebroplacental Ratio Thresholds: Superiority of ≤1.1 Over Less Than 1 for Predicting Adverse Perinatal Outcomes

**DOI:** 10.7759/cureus.92417

**Published:** 2025-09-16

**Authors:** Malvika Grover, Manisha Behal, Rupinder Singh

**Affiliations:** 1 Department of Obstetrics and Gynaecology, Maharishi Markandeshwar Medical College and Hospital, Kumarhatti, IND; 2 Department of Radiology, Maharishi Markandeshwar Medical College and Hospital, Kumarhatti, IND

**Keywords:** cerebroplacental ratio, doppler ultrasonography, fetal distress, intrapartum outcomes, perinatal morbidity

## Abstract

Background

The cerebroplacental ratio (CPR), calculated as the ratio of middle cerebral artery pulsatility index to umbilical artery pulsatility index, is a non-invasive Doppler marker reflecting placental function and fetal adaptation. While a CPR cut-off <1 has been traditionally used, emerging evidence suggests that ≤1.1 may provide superior predictive accuracy for adverse perinatal outcomes.

Objective

This study aimed to compare the predictive value of CPR cut-offs <1 and ≤1.1 in identifying term pregnancies at risk of intrapartum complications and adverse neonatal outcomes.

Methods

This prospective observational study was conducted at Maharishi Markandeshwar Medical College and Hospital, Kumarhatti, India, over 18 months, including 180 antenatal women with uncomplicated singleton pregnancies at 37-42 weeks. Doppler ultrasonography was performed to assess CPR, and outcomes were analyzed against the two cut-off values. Primary outcomes were intrapartum fetal heart rate abnormalities, mode of delivery, liquor characteristics, birth weight, and neonatal intensive care unit (NICU) admission.

Results

CPR <1 was significantly associated with abnormal fetal heart rate (100% vs. 18.6%; p=0.001), operative deliveries (100% vs. 20.3%; p=0.009), and NICU admissions (100% vs. 19.8%; p=0.001). When the threshold was raised to ≤1.1, predictive strength improved, with abnormal cardiotocography (CTG) (73.5% vs. 7.5%; p=0.001), higher emergency caesarean for fetal distress (100% vs. 84.6%; p=0.043), meconium-stained liquor (41.2% vs. 1.4%; p=0.001), and NICU admission (64.7% vs. 11%; p=0.001) all showing stronger associations.

Conclusion

CPR ≤1.1 outperforms the conventional cut-off of <1 in predicting adverse perinatal outcomes at term. Incorporating this threshold into routine antenatal surveillance may allow the earlier identification of at-risk fetuses and timely intervention to improve neonatal outcomes.

## Introduction

Early prediction of adverse feto-maternal outcomes remains a major challenge in modern obstetrics. Impaired fetal growth velocity and unfavorable consequences for both mother and fetus may occur even among fetuses that appear to have normal growth for gestational age [1]. Hence, fetal size alone is insufficient to predict adverse outcomes at term. In this context, the cerebroplacental ratio (CPR) has emerged as a valuable tool for identifying at-risk fetuses, including both small-for-gestational-age and appropriate-for-gestational-age fetuses, and for predicting adverse pregnancy outcomes [2].

CPR, the ratio of middle cerebral artery (MCA) to umbilical artery (UA) pulsatility indices, reflects placental and cerebral vascular resistance. In placental insufficiency, increased placental resistance and reduced cerebral resistance from the brain-sparing effect help maintain fetal oxygen and nutrient supply [3]. Fetal hypoxia can cause neonatal complications (seizures, metabolic issues, death) and long-term effects (cerebral palsy, attention-deficit/hyperactivity disorder (ADHD), cognitive deficits). During labor, uterine contractions reduce uteroplacental flow, lowering MCA pulsatility index values and indicating cerebral redistribution. As labor progresses, the fetus's tolerance to hypoxia declines, increasing intrapartum risk [4].

Placental insufficiency also increases the risk of stillbirth and hypoxic-ischemic encephalopathy. Growth-restricted fetuses with limited reserve are particularly vulnerable to intrapartum hypoxia and often require interventions [5]. Placental abnormalities further predispose term infants to cerebral palsy, with delivery delays resulting in a significant risk of brain damage and lifelong disability. Intrapartum surveillance and timely intervention, often through caesarean section, are essential for such compromised fetuses. However, emergency caesarean delivery in the context of acute fetal distress is associated with worse neonatal outcomes [6].

Fetal hypoxia activates compensatory mechanisms such as changes in fetal heart rate, blood pressure elevation, and redistribution of blood flow toward the brain, heart, and adrenal glands. These changes cause a decline in MCA resistance index due to vasodilatation under low oxygen tension. The brain-sparing phenomenon, while protective, offers only partial neuroprotection. Several studies indicate that children who exhibited brain-sparing in utero demonstrate lower IQ scores and impaired cognitive performance later in life [7].

Electronic fetal monitoring is the most widely used intrapartum screening tool for hypoxia but has poor specificity and predictive value. Identifying at-risk fetuses before labor remains difficult. Doppler studies of the placental and cerebral circulations provide a non-invasive method of detecting blood flow abnormalities. Normally, MCA pulsatility index remains higher than UA pulsatility index, keeping CPR above 1 in uncomplicated pregnancies. A reduced CPR reflects placental dysfunction and abnormal fetal circulatory adaptation, serving as a proxy marker of impaired growth [8].

Low CPR in late gestation has been strongly associated with stillbirth, increased perinatal loss, and adverse neonatal outcomes, independent of fetal weight. Thus, assessing CPR at term offers an opportunity to identify fetuses who fail to reach their genetic growth potential or are at risk of perinatal compromise. These fetuses warrant strict intrapartum surveillance and timely intervention to prevent adverse outcomes. Conventionally, CPR <1 has been considered abnormal, but emerging evidence suggests that a slightly higher threshold (≤1.1) may offer better sensitivity without compromising specificity. The present study aims to evaluate and compare the predictive value of CPR cut-offs <1 and ≤1.1 in identifying adverse intrapartum and neonatal outcomes among term pregnancies.

## Materials and methods

Study setting and design

The present study was conducted in the Department of Obstetrics and Gynaecology at Maharishi Markandeshwar Medical College and Hospital (MMMCH), Kumarhatti, India, after obtaining approval from the institute's Institutional Ethics Committee (IEC) (approval number: MMMCH/IEC/22/595). It was a single-center, prospective, observational, hospital-based study. Written informed consent in the vernacular language understood by the participant was obtained from all eligible women prior to enrolment.

Study period and sample size

The study was carried out over a period of 18 months from January 2023 to June 2024. A total of 180 antenatal women were included. The sample size was calculated for cross-sectional studies using the formula for the difference between two population means: $\text{n}=\frac{2\left[\left(\frac{\text{Z&alpha;}}{2}+\text{Z&beta;}\right)^{2}\times\text{P}\left(1&minus;\text{P}\right)\right]}{\left(\text{p₁}&minus;\text{p₂}\right)^{2}}$ [9]. With α set at 0.05 ($\frac{\text{Z&alpha;}}{2}$=1.96) and a power of 80% (Zβ=0.842), and using p1, p2, and pooled prevalence (P) from a similar study, the estimated minimum sample size was 141. Anticipating the dropout rate, 180 participants were finally recruited.

Selection of participants

All antenatal women between 37 and 42+0 weeks of gestation admitted to the Department of Obstetrics of MMMCH were considered for the study. Only uncomplicated singleton pregnancies within this gestational age range and presenting in vertex position were included. Women with gestational age less than 37 weeks or beyond 42 weeks, those in active labor with cervical dilatation greater than 5 cm, pregnancies with fetal congenital anomalies detected on ultrasonography, multiple gestations, intrauterine fetal demise, or fetuses with estimated weights falling into small- or large-for-gestational-age categories were excluded. Similarly, women planned for elective caesarean section, those with medical or obstetric complications during the index pregnancy, or those unwilling to participate were not enrolled in the study.

Data collection

Maternal socio-demographic and obstetric variables, including age, educational status, occupation, socioeconomic class, and gravidity, were recorded at admission using a structured proforma (see Appendices) and verified from antenatal records. Intrapartum outcomes were documented by continuous electronic fetal monitoring with cardiotocography (CTG), and fetal heart rate (FHR) patterns were classified according to the International Federation of Gynecology and Obstetrics (FIGO) 2021 guidelines as normal or abnormal [1]. Mode of delivery was noted as spontaneous vaginal, instrumental, or caesarean section, and the indication for caesarean delivery was specified (fetal distress or arrest of descent). At delivery, liquor characteristics (clear or meconium-stained) and liquor volume (adequate or inadequate) were assessed. Neonatal outcomes included birth weight, measured immediately after birth using a calibrated digital scale, and admission to the neonatal intensive care unit (NICU), with indication documented.

Doppler ultrasonography

Doppler ultrasonography was performed using Philips Affinity 70G and 50G machines (Philips Healthcare, Andover, Massachusetts, United States) with a 3-7 MHz curvilinear transducer. All scans were conducted by the principal investigator under supervision. Umbilical artery waveforms were obtained from a free loop of cord, while MCA waveforms were measured at the proximal third of MCA near its origin from the internal carotid artery, using color Doppler to visualize the circle of Willis. The insonation angle was maintained close to 0°, and 3-10 consecutive waveforms were recorded. Peak systolic velocity was auto-traced, and the CPR was calculated as $\frac{\text{MCA pulsatility index}}{\text{UA pulsatility index}}$. All Doppler ultrasonography was performed by the principal investigator under supervision to measure MCA and UA pulsatility indices, from which the CPR was calculated. CPR thresholds of <1 and ≤1.1 were applied for outcome comparisons.

Statistical analysis

Data were entered in Microsoft Excel (Microsoft Corporation, Redmond, Washington, United States) and analyzed using IBM SPSS Statistics for Windows, Version 27.0 (Released 2019; IBM Corp., Armonk, New York, United States). Quantitative variables were summarized as mean±standard deviation (SD), while categorical variables were expressed as frequency and percentage. Comparison of proportions between groups was performed using the chi-squared test or Fisher's exact test when appropriate. A p-value of <0.05 was considered statistically significant.

## Results

The mean maternal age of the study participants was 26.66±3.78 years. With respect to education, 106 (58.89%) women had received secondary education, 41 (22.78%) were graduates, 32 (17.78%) had completed only primary education, and one (0.56%) was illiterate. Regarding occupational status, 178 (98.89%) women were housewives, while only two (1.11%) were employed. In terms of socioeconomic status, 155 (86.11%) participants belonged to the upper middle class, 22 (12.22%) to the lower middle class, and only one each (0.56%) to the upper, upper lower, and lower classes. Assessment of obstetric history showed that 90 (50%) women were primigravida, 88 (48.89%) were gravida 2-4, and two (1.11%) were gravida ≥5 (Table 1).

**Table 1:** Distribution of study participants according to socio-demographic and obstetric variables Age is presented as mean±standard deviation (SD) in years. Other variables are expressed as frequency (percentage).

Variable	Domain	Number	Percentage
Age	Mean age	26.66±3.78 years	
Education status	Illiterate	1	0.56
	Primary	32	17.78
	Secondary	106	58.89
	Graduate	41	22.78
Occupation status	Housewife	178	98.89
	Employed	2	1.11
Socioeconomic status	Upper	1	0.56
	Upper middle	155	86.11
	Lower middle	22	12.22
	Upper lower	1	0.56
	Lower	1	0.56
Obstetric history	Primigravida	90	50
	Gravida 2-4	88	48.89
	Gravida ≥5	2	1.11

Among women with CPR <1, all three (100%) had abnormal CTG patterns, whereas in the CPR ≥1 group, 144 (81.35%) had normal CTG and 33 (18.64%) had abnormalities. This association was highly significant (p=0.001). With regard to the mode of delivery, none of the women with CPR <1 delivered vaginally, and all three (100%) required operative delivery (instrumental vaginal or lower segment caesarean section (LSCS)). In contrast, 141 (79.66%) women with CPR ≥1 delivered vaginally, while 36 (20.34%) required operative delivery (p=0.009). Looking specifically at indications for LSCS, fetal distress was the leading cause in both groups, accounting for three (100%) of LSCS in CPR <1 and 33 (91.67%) in CPR ≥1, with no significant difference (p=0.602). Arrest of descent was reported in three (8.33%) cases with CPR ≥1. Regarding amniotic fluid characteristics, meconium-stained liquor was observed in one (33.33%) woman with CPR <1 compared to 15 (8.47%) with CPR ≥1, though this difference did not reach statistical significance (p=0.134). Similarly, inadequate liquor volume was noted in one (33.33%) woman with CPR <1, whereas all 177 (100%) with CPR ≥1 had adequate liquor. This association was highly significant (p=0.001) (Table 2).

**Table 2:** Association between CPR (CPR <1 and CPR ≥1) and intrapartum outcomes Indication of LSCS is shown only for women who underwent caesarean section. P-values are calculated using the chi-squared test; Fisher's exact test is applied when the expected frequency is <5. * indicates that p<0.05 (considered statistically significant). CPR: cerebroplacental ratio; CTG: cardiotocography; NVD: normal vaginal delivery; FD: fetal distress; MSL: meconium-stained liquor; LSCS: lower segment caesarean section

Variable	Domain	CPR <1		CPR ≥1		P-value
		Number	Percentage	Number	Percentage	
CTG	Normal	0	0	144	81.35	0.001*
	Abnormal	3	100	33	18.64	
Mode of delivery	NVD	0	0	141	79.66	0.009*
	Operative delivery (instrumental vaginal+LSCS)	3	100	36	20.34	
Indication of LSCS	FD	3	100	33	91.67	0.602
	Arrest of descent	0	0	3	8.33	
Liquor color	Clear	2	66.67	162	91.53	0.134
	MSL	1	33.33	15	8.47	
Liquor volume	Adequate	2	66.67	177	100	0.001*
	Inadequate	1	33.33	0	0	

Among women with CPR ≤1.1, nine (26.47%) had normal intrapartum FHR patterns, while 25 (73.52%) showed abnormal patterns. In contrast, 135 (92.47%) women with CPR >1.1 had normal FHR, with abnormalities noted in only 11 (7.53%). This association was highly significant (p=0.001). Regarding the mode of delivery, 25 (73.53%) women with CPR ≤1.1 required operative delivery (instrumental vaginal or LSCS), while only nine (26.47%) delivered vaginally. Conversely, 132 (90.41%) women with CPR >1.1 had spontaneous vaginal delivery, and 14 (9.58%) required operative intervention (p=0.001). In terms of indications for LSCS, all 25 (100%) cases in the CPR ≤1.1 group were due to fetal distress, whereas in the CPR >1.1 group, 11 (78.57%) were for fetal distress and 3 (21.43%) were for arrest of descent, showing a significant association (p=0.016). For amniotic fluid characteristics, meconium-stained liquor was substantially more frequent in CPR ≤1.1 pregnancies (14 (41.18%)) compared to CPR >1.1 (two (1.37%)), while clear liquor was observed in 20 (58.82%) and 144 (98.63%), respectively (p=0.001). With respect to liquor volume, one (2.94%) case with CPR ≤1.1 had inadequate liquor, whereas all 146 (100%) with CPR >1.1 had adequate liquor (p=0.038) (Table 3).

**Table 3:** Association between CPR (CPR ≤1.1 and CPR >1.1) and intrapartum outcomes Indication of LSCS is shown only for women who underwent caesarean section. P-values are calculated using the chi-squared test; Fisher's exact test is applied when the expected frequency is <5. * indicates that p<0.05 (considered statistically significant). CPR: cerebroplacental ratio; FHR: fetal heart rate; NVD: normal vaginal delivery; FD: fetal distress; MSL: meconium-stained liquor; LSCS: lower segment caesarean section

Variable	Domain	CPR ≤1.1		CPR >1.1		P-value
		Number	Percentage	Number	Percentage	
Intra partum FHR pattern	Normal	9	26.47	135	92.47	0.001*
	Abnormal	25	73.52	11	7.53	
Mode of delivery	NVD	9	26.47	132	90.41	0.001*
	Operative delivery (instrumental vaginal+LSCS)	25	73.53	14	9.58	
Indication of LSCS	FD	25	100	11	78.57	0.016*
	Arrest of descent	0	0	3	21.43	
Liquor color	Clear	20	58.82	144	98.63	0.001*
	MSL	14	41.18	2	1.37	
Liquor volume	Adequate	33	97.06	146	100	0.038*
	Inadequate	1	2.94	0	0	

A significant association was observed between CPR and neonatal birth weight. All three (100%) neonates in the CPR <1 group had low birth weight (<2500 g). In the CPR ≥1 group, 24 (13.56%) neonates weighed <2500 g, 152 (85.88%) had normal birth weight (≥2500 to <4000 g), and one (0.56%) was macrosomic (≥4000 g). This difference was statistically significant (p=0.001). Similarly, a strong correlation was noted with NICU admissions. All three (100%) neonates in the CPR <1 group required NICU admission, compared to 35 (19.77%) in the CPR ≥1 group. The majority, 142 (80.23%), of neonates with CPR ≥1 did not require NICU care. This association was also highly significant (p=0.001) (Table 4).

**Table 4:** Association between CPR (CPR <1 and CPR ≥1) and neonatal outcomes P-values are calculated using the chi-squared test; Fisher's exact test is applied when the expected frequency is <5. * indicates that p<0.05 (considered statistically significant). CPR: cerebroplacental ratio; NICU: neonatal intensive care unit

Variable	Domain	CPR <1		CPR ≥1		P-value
		Number	Percentage	Number	Percentage	
Birth weight	<2500 grams	3	100	24	13.56	0.001*
	≥2500 to <4000 grams	0	0	152	85.88	
	≥4000 grams	0	0	1	0.56	
NICU admission	Yes	3	100	35	19.77	0.001*
	No	0	0	142	80.23	

A statistically significant association was observed between CPR and birth weight. In the CPR ≤1.1 group, 10 (29.41%) neonates had low birth weight (<2500 g), compared to 17 (11.64%) in the CPR >1.1 group. The majority of neonates in both groups weighed between ≥2500 and <4000 g (24 (70.59%) vs. 128 (87.67%), respectively), while only one (0.68%) neonate in the CPR >1.1 group had a birth weight ≥4000 g. This difference was statistically significant (p=0.030). A highly significant relationship was also noted between CPR and NICU admission. In the CPR ≤1.1 group, 22 (64.71%) neonates required NICU admission, compared to 16 (10.96%) in the CPR >1.1 group. Conversely, 130 (89.04%) neonates in the CPR >1.1 group did not require NICU care, whereas only 12 (35.29%) in the CPR ≤1.1 group avoided admission. This difference was highly significant (p=0.001) (Table 5).

**Table 5:** Association between CPR (CPR ≤1.1 and CPR >1.1) and neonatal outcomes P-values are calculated using the chi-squared test; Fisher's exact test is applied when the expected frequency is <5. * indicates that p<0.05 (considered statistically significant). CPR: cerebroplacental ratio; NICU: neonatal intensive care unit

Variable	Domain	CPR ≤1.1		CPR >1.1		P-value
		Number	Percentage	Number	Percentage	
Birth weight	<2500 grams	10	29.41	17	11.64	0.030*
	≥2500 to <4000 grams	24	70.59	128	87.67	
	≥4000 grams	0	0	1	0.68	
NICU admission	Yes	22	64.71	16	10.96	0.001*
	No	12	35.29	130	89.04	

## Discussion

The most appropriate cut-off value of the CPR for predicting adverse perinatal outcomes remains debatable. Researchers have proposed different thresholds, including absolute values (<1, <1.08, or <1.1), centile-based cut-offs (<5th or 10th centile), or multiples of median (MoM). In our study, when the CPR cut-off was taken as 1.1, abnormal CTG was significantly more common in women with CPR ≤1.1 (73.52%) compared to CPR >1.1 (7.53%). Similar findings were reported in Poland, where 62.3% of women with CPR <1.1 had abnormal CTG compared to 19% with CPR ≥1.1 [10]. When a cut-off of 1 was applied, all women with CPR <1 (100%) had abnormal CTG compared to 18.64% in the CPR ≥1 group, in line with previous reports that demonstrated a strong association between low CPR and intrapartum CTG abnormalities [10]. These findings support the strong predictive value of low CPR for intrapartum fetal distress.

With respect to mode of delivery, our results demonstrated a markedly higher rate of caesarean section in women with CPR ≤1.1 (73.53%) compared to those with CPR >1.1 (8.9%). These observations are consistent with studies from Suez Canal University Hospital (40% vs. 25.6%) [4], Ambedkar Hospital (87.5% vs. 23.53%) [11], and Egypt, where higher LSCS rates were reported in the low CPR group [12]. Similarly, when the CPR cut-off was taken as 1, our study revealed a 100% LSCS rate in women with CPR <1 compared to 19.77% in CPR ≥1.

Emergency LSCS for fetal distress also correlated strongly with low CPR. In our study, 100% of women with CPR ≤1.1 required LSCS for intrapartum distress, compared to 84.61% in those with CPR >1.1. Similar findings were reported by studies from Suez Canal University Hospital (40% vs. 9.6%) [4] and Ambedkar Hospital (87.5% vs. 23.53%) [11], reinforcing the role of CPR as a predictor of emergency intervention.

The association between CPR and NICU admission was also evident. In our study, 64.7% of neonates from mothers with CPR ≤1.1 required NICU admission, compared to only 10.9% in CPR >1.1. Birth weight was another important outcome associated with CPR. A study from Tamil Nadu also demonstrated that neonates of mothers with low CPR values had significantly lower birth weights [13]. 

Birth weight was another important outcome associated with CPR. Studies from Tamil Nadu [13], Egypt [12], and our study consistently demonstrated that neonates from mothers with lower CPR values had significantly lower birth weights. In our cohort, 29.4% of neonates in the CPR ≤1.1 group weighed less than 2.5 kg, compared to 11.6% in the CPR >1.1 group.

The effect of CPR on APGAR scores was variable across studies. At Suez Canal University Hospital, CPR ≤1.1 was associated with significantly reduced APGAR scores at one and five minutes [4]. A study from Egypt demonstrated lower APGAR at five minutes in neonates with CPR <1 [14]. However, in our study, there was no statistically significant difference in APGAR scores between low and normal CPR groups.

Overall, our findings align with previous research indicating that low CPR is a strong predictor of adverse intrapartum and perinatal outcomes, including abnormal CTG, increased LSCS rates, emergency LSCS for fetal distress, NICU admissions, and low birth weight. However, its predictive value for APGAR scores appears less consistent. These observations support the utility of CPR as a simple, non-invasive Doppler parameter to guide antenatal surveillance and timely intervention in high-risk pregnancies.

The present study has certain limitations. First, being a single-center study with a relatively modest sample size, the generalizability of our findings may be limited. Second, only term, uncomplicated singleton pregnancies were included, which may restrict applicability to high-risk or preterm populations. Third, Doppler measurements were performed by a single investigator, which, although ensuring consistency, did not allow the assessment of inter-observer variability. Lastly, long-term neonatal outcomes were not assessed; hence, the predictive value of CPR beyond the immediate perinatal period could not be evaluated. 

## Conclusions

CPR is a simple, non-invasive tool that may help identify at-risk pregnancies and guide delivery planning. In this study, CPR <1 was linked to abnormal intrapartum FHR, reduced liquor, increased operative deliveries, low birth weight, and NICU admission. Using a threshold of CPR ≤1.1 detected additional adverse outcomes, such as higher LSCS rates for fetal distress and meconium-stained liquor. Women with CPR >1.1 generally experienced favorable outcomes, while CPR ≤1.1 indicated a need for closer monitoring and possible referral to higher-level care. Although our study was limited by its single-center design and modest sample size, CPR ≤1.1 may be considered a better predictor of adverse perinatal outcomes. Future multicenter studies with larger and more diverse cohorts are essential to validate these findings, enhance external generalizability, and ultimately improve the management of high-risk pregnancies to minimize adverse perinatal outcomes.

## Appendices

**Table 6:** Structured proforma used in the study CTG: cardiotocography; NICU: neonatal intensive care unit; PI: pulsatility index; CPR: cerebroplacental ratio

Domain	Variable	Response/options
Maternal socio-demographic details	Name/study ID	
	Age (years)	
	Educational status	Illiterate/primary/secondary/graduate/postgraduate
	Occupation	Housewife/employed
	Socioeconomic class	Upper/upper middle/lower middle/upper lower/lower
Obstetric history	Gravidity	
	Parity	
	Gestational age at admission (weeks)	
	Presentation	
	Previous obstetric complications	Yes/no (if yes, specify)
Intrapartum outcomes	CTG	Normal/abnormal
	Mode of delivery	Normal vaginal/instrumental/caesarean
	Indication for caesarean	
	Liquor characteristics	Clear/meconium-stained
	Liquor volume	Adequate/inadequate
Neonatal outcomes	Birth weight (grams)	
	APGAR score (1 min/5 min)	
	NICU admission	Yes/no
	Indication for NICU admission	
Doppler findings	Umbilical artery PI	
	Middle cerebral artery PI	
	CPR	
	Category	<1/≤1.1/>1.1
